# WhatsApp: a supplementary tool for improving bed nets universal coverage campaign in Mozambique

**DOI:** 10.1186/s12913-019-3929-0

**Published:** 2019-02-01

**Authors:** Jorge A. H. Arroz, Baltazar Neves Candrinho, Figueiredo Mussambala, Marta Chande, Chandana Mendis, Sónia Dias, Maria do Rosário O. Martins

**Affiliations:** 1 World Vision International, Maputo, Mozambique; 2 National Malaria Control Programme, Maputo, Mozambique; 3 Global Health and Tropical Medicine, GHTM, Instituto de Higiene e Medicina Tropical, IHMT, Universidade Nova de Lisboa, UNL, Rua da Junqueira 100, 1349-008 Lisbon, Portugal

**Keywords:** WhatsApp, Mentoring, Qualitative research, Universal coverage bed nets campaign, Mozambique

## Abstract

**Background:**

WhatsApp (WA) is the most recent and attractive applicative among Smartphone users. The use of WA in healthcare environment has been shown of multiple benefices. Mozambique team involved in 2017 bed nets universal coverage campaign (UCC) implemented a distant mentoring strategy using WA. This study aims to perform a descriptive analysis of the use of WA as a supplementary tool for mentoring provincial and district health teams during bed nets universal coverage campaign in Mozambique.

**Methods:**

Using WA, a qualitative study was carried out between March and July 2017. Seven WA groups were created. One group for central-level team, and six groups corresponding to each implementation province. The WA content was analyzed, grouped into separate themes, and subject to information triangulation among researchers and group participants. Saturation guided the quantity and quality of information.

**Results:**

A total of 511 members were included in all WA groups. Of these, 96% were provincial WA groups. A total of 24,897 messages (text and images) were exchanged in all WA groups. The main communication form was text (22,660–91%), followed by images (2237–9%). Five themes emerged from content analyses: 1) administrative/financial, 2) logistic, 3) planning and implementation, 4) monitoring and evaluation, and 5) best practice.

**Conclusions:**

The use of WA during universal coverage bed nets campaign implementation in Mozambique fostered central-level coordination, providing implementation support to district and provincial teams, and promoting wider and timely information sharing among group members.

## Background

WhatsApp (a pun on the phrase What’s Up) is probably the most recent and attractive applicative among Smartphone users to text, exchange photos, videos and voice note [1]. The use of mobile applications has been shown to be useful for educational purpose [2–5]. Using WhatsApp (hereinafter referred to as WA) in the primary health care education setting has demonstrated a number of benefits for undergraduate nurses. These include the usefulness of the application for integrating theory and clinical practice; increasing the availability of resources for test preparation and providing a platform for clarification of uncertain aspects of the course [6].

Mozambique team involved in 2017 bed nets universal coverage campaign (hereinafter referred to as UCC) developed multifaceted implementation strategies to increase UCC implementation performance, access and demand of long-lasting insecticidal nets (LLINs). Flash mentoring was a one of the strategies used. Flash mentoring is defined by us in the UCC context as a meeting or discussion that enables individuals (district team) to learn and seek guidance from a more experienced person (provincial or central team) who can transfer knowledge and experience on relevant aspects of the UCC. The purpose is to provide a continuous learning opportunity (mentoring) and technical support with unlimited commitment of time and to ensure a smooth learning and transition from initial training to field implementation. The platform considered for this continuous mentoring process was a WA messenger applicative. The choice of WA was based on the central team perception of being commonly used among health professionals in Mozambique, and with its group communication feature, interaction is perceived as being practical.

This study aims to perform a descriptive analysis of the use of instant messaging communication (WA) as a supplementary tool for mentoring provincial and district health teams during bed nets universal coverage campaign in Mozambique.

## Methods

### Study design

A descriptive qualitative study was carried out between March and July 2017. Seven UCC WA groups were created involving central, provincial and district health teams. The first UCC WA group was created for central-level health team. The other six UCC WA groups were created for each province, namely: Niassa, Cabo Delgado, Zambezia, Tete, Manica, and Sofala.

### Inclusion criteria

The following inclusion criteria were used to select members for the WA groups: (i) being part of the national, provincial and district coordination groups; (ii) having a cell phone with WA already installed; (iii) being familiarly with WA features; and (iv) voluntary being able to participate in the WA group.

### WhatsApp group composition and purpose

Central-level WA group was composed of National Malaria Control Programme personnel and civil society partners. These personnel were involved in coordination of the UCC, providing technical, logistic, communication, and monitoring and evaluation support to all implementing provinces. The purpose of this WA group was to establish a core coordination and communication central-level group.

Provincial WA groups were composed of provincial and district health authorities of each province. The provincial health authorities were: provincial medical chief, head of the public health department, malaria focal point, and supervisors of each district. The district health authorities were: district health director, district medical chief, malaria focal point, community involvement focal point, data analysis focal point, and district warehouse keeper. All central-level WA group members were also members of the provincial WA groups. The purpose of these provincial UCC WA groups were to provide a continuous learning opportunity (mentoring) and technical support with unlimited commitment of time and to ensure a smooth learning and transition from initial training to field implementation. Table 1 summarizes the WA strategy. All WA group members voluntary agreed in making part of the groups as a way to interact during the campaign implementation, and gave their verbal consent after explanation of the group creation objectives.

**Table 1:** Specification of the strategy: mentoring using WA Messenger

Domain	WA Messenger
Actor(s)	Central-level health team (National Malaria Control Programme and civil society partners), Provincial and District Health Teams from Niassa, Cabo Delgado, Zambezia, Tete, Manica and Sofala provinces
Action	Mentoring using WA Messenger
Target(s) of the action	Provincial and District Health Teams from Niassa, Cabo Delgado, Zambezia, Tete, Manica and Sofala provinces
Temporality	March to July 2017
Dose	Whenever necessary, involving all participants in the WA group
Justification	Pragmatic justification: to provide a continuous learning opportunity (mentoring) and technical support with unlimited commitment of time and to ensure a smooth learning and transition from initial training to field implementation.

### Context

Mozambique sits on the southeast coast of Africa, with a population of 27,977,863 as of 2015 [7]. The 2015 Human Development Index put Mozambique at the bottom of the ranking (180th out of 188 countries and territories) [7]. Malaria remains the most common cause of death, responsible for 35% of child mortality and 29% for the general population [7]. Cell phones penetration rates among households in Mozambique have increased from 23.5% in 2008/9 to 55.8% in 2014/15 [8]. This cell phones penetration rate is greater in urban than rural areas, covering 78.8 and 45.6% of households, respectively [8]. Smartphone penetration rate among health professionals is unknown; however there are strong impressions that the majority of health professionals has and makes use of different types of Smartphone.

### Data collection

Using the technique of free (unstructured) and participant group observation (researchers were also members of the WA groups as central-level team members), researchers collected data (messages, images) from all WA groups. The observation was on UCC related exchanged messages and images, which were the focus of the remote mentoring process. This technique allowed immersion of the researchers in the WA settings, offering the opportunity to learn directly from the experience and to provide mentoring to provincial and district teams. The first observation started on February 28th 2017, with the central-level WA group. The observation ended on July 31st 2017.

### Potential bias and techniques to enhance trustworthiness

The fact that researchers were members of the WA groups could be seen as a potential bias. However, although this might be a valid concern, and in order to minimize potential researchers bias, the active participation of the researchers was: (i) as mentors (the main objective of the evaluation), clarifying questions raised by implementers; (ii) raising questions about campaign implementations status (monitoring). All the researchers had background training on active listening technique, which was of most value for interaction during the observation period.

### Data analysis

Related to UCC data from all WA groups were extracted and exported to Notepad Version 6.1 (Npad 6.1). From Npad 6.1, data were exported to Microsoft Office Excel 2007 for quantitative analysis purpose, namely: size of each WA group, type of communication (text, images), the degree of participation from each WA group.

The same exported documents to Npad 6.1 were also subject of qualitative analysis, initially manually, then with support of WhatsApp Analyzer Version 2.6.8 applicative and the qualitative data analysis and research software ATLAS.ti 8 – Windows. The content of the conversation were subject to classic content analysis, looking for themes (based on frequencies, common word search, identification and classification of themes) and semantic (connections between themes in the text). The content analysis was performed by all researchers, looking globally (not at individual-level), and being focused on campaign-related exchanged messages and images, i.e., messages none related with UCC where excluded from the analysis (were previously deleted from all WA groups by the researchers, before being exported to Npad 6.1). This information was grouped into separate emerging themes and was initially subject to triangulation among researchers, and then the researchers performed a triangulation with two provincial-level participants of each WA group, in a public meeting held by National Malaria Control Programme. Saturation guided the quantity and quality of information analysis.

## Results

### WhatsApp groups features and type of communication

A total of 511 members were included in all WA groups. Of these, 96% were members of the provincial WA groups. Tete and Zambezia WA groups had the higher percent of members, with 24 and 22% of total members, respectively. The first WA group (central-level) was created in February 28, 2017; the last WA group was created in May 24, 2017.

A total of 24,897 messages (text and images) were exchanged in all WA groups. The main communication form was text (22,660–91%), followed by images (2237–9%). Tete and Central-level WA groups were the most active in texting, with 20.6 and 19.6% of total text messages sent, respectively. Sofala and Central-level WA groups were the most active in sending images, with 27.4 and 21.5% of total images sent – Table 2.

**Table 2:** WhatsApp groups creation dates, members, and type of communication during bed nets universal coverage campaign in Mozambique - 2017

Whatsapp groups	Creation date	Members		Text messages		Image messages		Messages (text plus images)	
		*N*	%	*N*	%	*N*	%	*N*	%
Central-level	28-Feb-17	19	3.7	4450	19.6	480	21.5	4930	19.8
Cabo Delegado	8-Mar-17	62	12.1	4242	18.7	201	9.0	4443	17.8
Zambezia	23-Mar-17	114	22.3	3505	15.5	422	18.9	3927	15.8
Niassa	26-Mar-17	57	11.2	1322	5.8	78	3.5	1400	5.6
Sofala	23-May-17	53	10.4	1550	6.8	614	27.4	2164	8.7
Tete	24-May-17	122	23.9	4660	20.6	323	14.4	4983	20.0
Manica	24-May-17	84	16.4	2931	12.9	119	5.3	3050	12.3
Total provincial WhatsApp group		492	96.3	18,210	80.4	1757	78.5	19,967	80.2
Total provincial and central-level WhatsApp group		511	100.0	22,660	100.0	2237	100.0	24,897	100.0

### Emerged themes during content analysis

Five themes emerged from content analyses: 1) administrative/financial, 2) logistic, 3) planning and implementation, 4) monitoring and evaluation, and 5) best practice.

#### Theme 1: Administrative/financial

The dominant theme identified was the use of WA for information sharing and questions about administrative procedures and financial aspects during implementation process, i.e., sending list of bank accounts of the district personnel, asking subsidies amount involved in a particularly phase of the campaign, sharing payments delays or others delays related to the administrative and financial implementation process:

*“Good evening… I would like to know about the payment process during the distribution phase for those using bank accounts…” (Zambezia member, WA group Zambezia).*

*“Important: … all campaign intervenient should know how much will receive before the beginning of the activity…” (central-level member, WA group Niassa).*

*“Attention: all the trainers are from health or education sector… bank transfer is advisable” (central-level member, WA group Cabo Delgado).*

#### Theme 2: Logistic mentoring and technical support

**Fig. 1:**
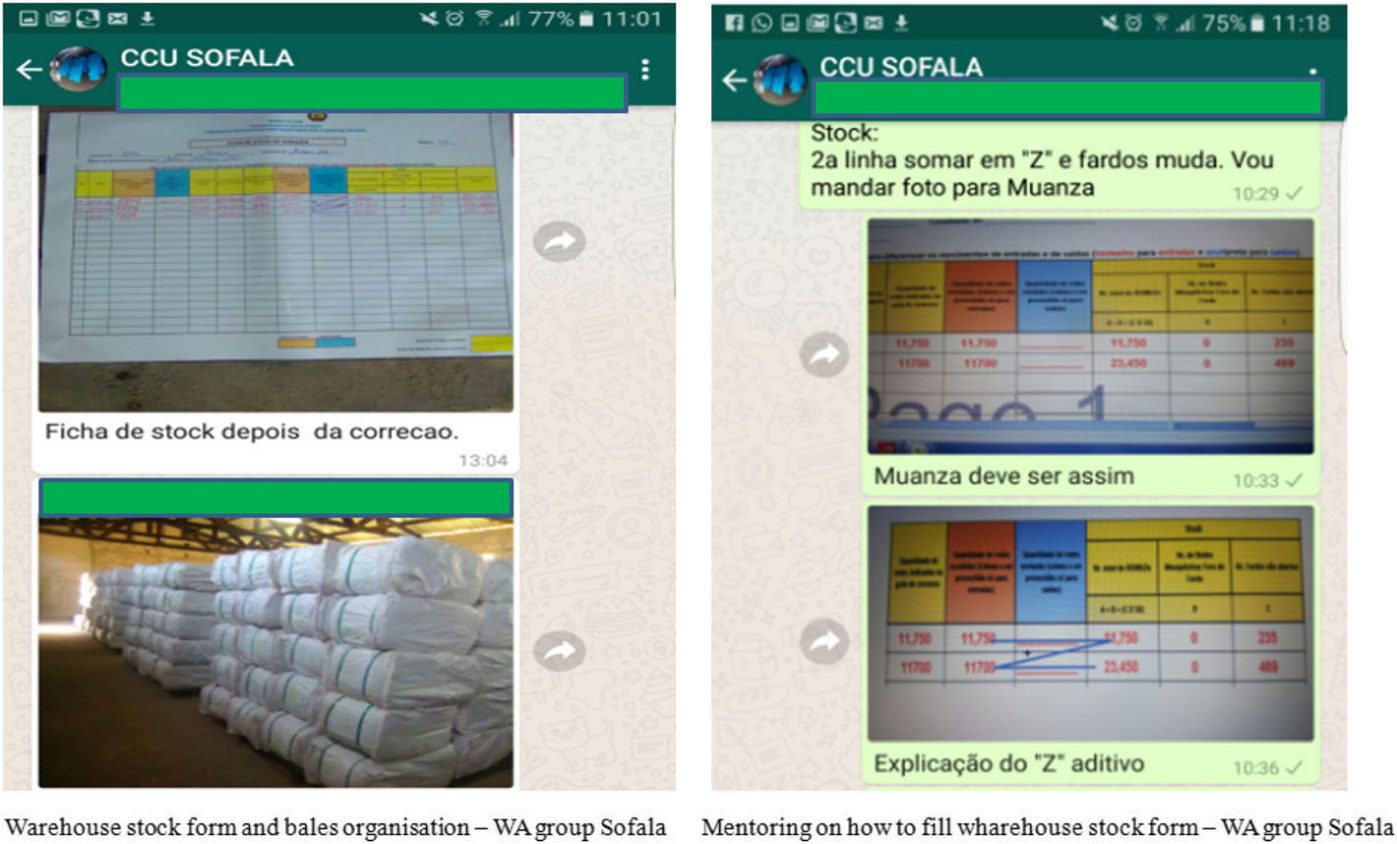
Examples of images shared - warehouse stock form, bales organisation, and mentoring process on how to correctly fill the warehouse stock form

Logistic theme was mainly related to three aspects: sharing images of filled warehouse stock form, sharing images about bed nets bales organization in the warehouse, and campaign materials received/distributed or additional needs. Information about bed nets needs and stocks after distribution were also shared. Technical support and guidance about these aspects was timely provided – Fig. 1.

*“Districts from round II will start distribution on September 25th. Reason: reverse logistics of the remaining bed nets from first round districts to reinforce the districts of round II…” (central-level member, WA group central level).*

*“[district name] has 151 bales and according to the plan we need 131 bales for tomorrow. Just in case we request 50 additional bales…” (Niassa member, WA group Niassa).*

*“We have information that some districts began to receive the bed nets last week, but we still do not have confirmation from the districts, with the exception of [district name]”(Tete member, WA group Tete).*

*“Good morning, the district of [district name] asked for backup bed nets to cover the unregistered population, so we must first analyze this case at the meeting” (central-level member, WA group Tete).*

#### Theme 3: Planning and implementation

Planning and implementation messages were related to central-level coordination about trainings and support supervision at provincial and district levels, harmonization of technical support, questions from district teams about doubts raised during district trainings, supervision, or findings in the field and how to proceed. Images were related to sharing evidence of training process, household registration and distribution, adjustments of the timeline, difficulties founded in the field, and others planning and implementation aspects and findings in “real world”:

*“It seems there is some misunderstanding in the timeline… we need to harmonize today.” (central-level member, WA group central level).*

*“Good morning. Following the timeline question…. Manica province should follow as planned; Tete and Sofala should follow according to the sequence of the standard budget…” (central-level member, WA group central level).*

*“… during investigation we found more than 30 coupons…. We suspected of fraud… the household registrar confessed the fraud… we channeled this issue to the local authorities…” (Niassa member, WA group Niassa).*

*“Good day. Please remember that the cap is 4 bed nets per household” (central-level member, WA group Zambezia, Niassa, Cabo Delgado).*

#### Theme 4: Monitoring and evaluation – Feedback

**Fig. 2:**
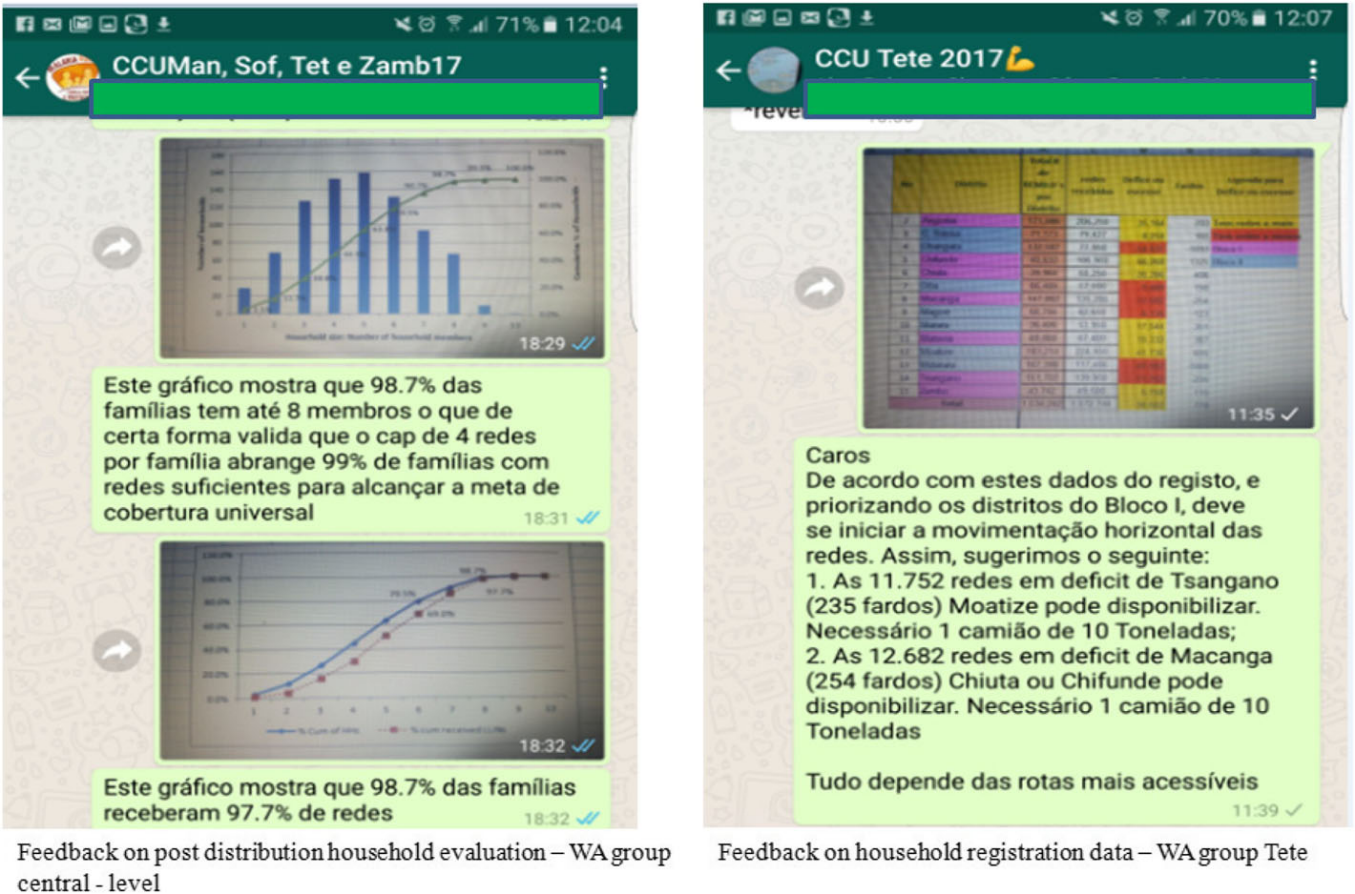
Examples of images shared for monitoring and evaluation technical support

Data monitoring and evaluation messages and images happened in two main phases: household registration and distribution. During these phases, daily report of data collected occurred, and provincial and central teams provided feedback – Fig. 2.

*“Three days cumulative information, number of households – 356,390, retrieved coupons – 356,390, distributed bed nets – 896,411, household coverage – 64%, percentage of distributed bed nets 69%” (Manica member, WA group Manica).*

*“First day coverage, distributed 9,121 bed nets – 47.2%, households 3,904 – 43%.... are these data correct?...” (central - level member, WA group Cabo Delgado).*

*“[district name] second day: population: 18,886; households = 1,038; coupons = 1,038; stickers = 636; bed nets needs = 2,809; …. The discrepancies on the stickers were due to stock out of stickers in the headquarter of the district. We still have no data from [locality name] due to lack of communication with the registrar.” (Cabo Delgado member, WA group Cabo Delgado).*

*“[district name] had the coverage of 96% of delivered bed nets and is collecting the remaining bed nets in the satellite warehouses to have the real stock” (Niassa member, WA group Niassa).*

#### Theme 5: Best practice

Best practice information was shared within and between WA groups:

*“[district name] can share your experience for timely accomplish planned activities?” (Tete member, WA group Tete).*

*“… If there are difficulties with localities authorities, you should address the difficulties with the district authorities…” (Tete member, WA group Tete).*

*“I am sharing the distribution plan… I had difficulties in defining how many supervisors, but according to micro-planning my district has 5 supervisors…” (Zambezia member, WA group Zambezia).*

*“I am sharing some coupons fraud attempts reported by other provinces… the objective is to learn…. Remaining coupons and stickers used during household registration phase must be collected and returned… remember that coupons have several items to be checked for possible fraud attempts…” (central - level member, WA group Zambezia).*

## Discussion

The results of this study shows that WA is a useful tool to foster central-level coordination (4450 text messages and 480 images related to bed nets UCC) and to provide implementation support (mentoring) during UCC in Mozambique - total of 24,897 messages (22,660 text-type and 2237 images-type) related to the following UCC themes: administrative/financial, logistic, planning and implementation, monitoring and evaluation, and best practice.

The reason for sending more text messages than images may be related to the relatively higher costs of uploading or downloading images. Texting is easier, gives more details on how to proceed, and is cheaper than sending images. WA group members remained actively engaged throughout the life period of the campaign, exchanging information about the implementation process in their districts and provinces.

Coordination, planning, communication and information sharing are crucial in the success of a bed nets distribution campaign. Using WA, Malaria consortium in Uganda reported successful coordination, quicker decision-making, and information sharing during the 2017 campaign [9]. Chawla et al. [10] reported WA group high acceptability amongst users for mentoring, correct compilation of stock reports, reminder for reports, and stock/consumption reporting channel. Others studies and reports in the medical field have shown that WA increases student participation, enhance the feedback process and improve communication between student and tutor [2–5]. A number of other WA benefits (e.g. improved clinical practice, clarification of uncertain aspects) have been demonstrated in the primary health care education setting [6]. Studies into the use of WA in the healthcare environment have demonstrated the effectiveness in its use as a communication tool, by overcoming human factor barriers to effective communication [2–6, 9, 11]. It also allows for increased connectedness between team members [2–6, 11].

The domination of administrative/financial themes in this study was probably related to information sharing gaps during initial training of district teams. This happened because the initial training time process was reduced from 10 to 6 days; therefore administrative/financial aspects were relegated to the second plan during the initial training. Mismatch between programmatic and financial implementation could also contributed since there are very strict criteria for administrative and paying procedures. These criteria were not timely followed, leading to gaps and raising payment constrains shared in WA groups.

Logistics aspects (bed nets control and tracking procedures), planning and implementation also dominated the groups’ interaction since they are critical aspects in universal coverage bed nets campaign. The first theme is often subject to accountability, and the second often subject to questions related to implementation fidelity (the degree to which an intervention is delivered as intended [12] - also being subject to accountability) and the main goal of the process: to ensure that at least 90% of households has sufficient bed nets in order to achieve universal coverage target, i.e., one bed net for every two persons. The other themes (monitoring and evaluation, and best practice) emerged as a support of the logistics and planning and implementation themes.

The absence of face-to-face interaction between central – provincial – district teams during implementation process may be seen as a potential cons by using WA. In fact, a certain tendency to reduce face-to-face meetings was perceived. This tendency should be carefully explored since in this study, multiple tasks, campaign dedicated staff shortage, and the fact of being running out the campaign simultaneously in at least three provinces could have played a significant role in this face-to-face perception of meeting reduction.

### Limitations

The study does not compare the use of WhatsApp with the use of any other means of communications. The presence of a ‘case’ without a ‘control’ can be seen as potential limitation. However, the study objective is not to compare different means of communication; the objective is to describe a supplementary communication tool with potential benefits in coordination and mentoring.

### Future implications

WhatsApp is a Web 2.0 application (i.e., enables many-to-many flow of information) and was used in this case as public health 2.0 intervention, offering an opportunity for all stakeholders to actively take part and collaborate on a health issue – UCC in this case. With this in mind, WA use can be future explored to foster, strength, and improve malaria programmes (and other health programmes) management and service delivery. It can also be seen as a valuable supplementary tool during implementation of the Global Technical Strategy for Malaria 2016–2030.

## Conclusions

The use of WA Messenger applicative during bed nets universal coverage campaign implementation in Mozambique fostered central-level coordination, provided implementation support to district and provincial teams, and promoted wider and timely information sharing among group members. The use of WA can be further explored in terms of continuous monitoring of post bed nets distribution communication and mobilization activities, and for general malaria control and elimination activities amongst implementers’ partners. Using comparison group and performing qualitative examination of perceptions (interviews or focus groups for example) and analysis of those not using WA is herein suggested as next steps to prove the feasibility and acceptability of WA during bed nets universal coverage campaign.

## Abbreviations

LLINs: Long-Lasting Insecticidal Nets
UCC: Universal Coverage Campaign
WA: WhatsApp
